# Lights Out! The Body Needs Sleep: Electronic Devices and Sleep Deficiency

**DOI:** 10.7759/cureus.9292

**Published:** 2020-07-20

**Authors:** Nicholas Tsouklidis, Nayibeth Tallaj, Yinabeth Tallaj, Stacey E Heindl

**Affiliations:** 1 Medicine, California Institute of Behavioral Neurosciences and Psychology, Fairfield, USA; 2 Health Care Administration, University of Cincinnati Health, Cincinnati, USA; 3 Medicine, Atlantic University School of Medicine, Gros Islet, LCA; 4 Pediatrics, Flushing Hospital Medical Center, Flushing, USA; 5 Internal Medicine, Flushing Hospital Medical Center, Flushing, USA; 6 Internal Medicine, Universidad Iberoamericana (UNIBE), Santo Domingo, DOM

**Keywords:** sleep hygiene, rem, rem latency, childhood obesity, sleep disorder, narcolepsy, behavioral changes, electronic devices, mental health, insomnia

## Abstract

Sleep hygiene in children and young adults has been a topic of interest in scientific studies geared towards understanding metabolism, mental health, neuroscience, and in reference to the quality of life. There are multiple factors that may contribute to poor sleep hygiene in children, many of these include environmental factors and genetic components. This review article will pay particular focus on environmental factors which as of late, have been increasing contributors to poor sleep hygiene in children. Ultimately, these factors lead to unhealthy habits that transform into unhealthy lifestyles in younger populations worldwide. This article will concentrate on studies conducted in the United States, Canada, Switzerland, Norway, and Belgium. In each of these studies, children who are exposed to increased use of electronic devices such as tablet computers, television, desktop computers, and other mobile devices during the late hours of the night, are evaluated and assessed for changes in their rapid eye movement (REM) sleep cycles, sleep latency, body mass index (BMI) levels, obesity risks, and other neurologic deficits which may be linked to this inappropriate use of technology during peak hours of the night. We will understand the physiology behind how sleep works, the events leading up to sleep, and disruptions that can occur, and their devastating effects.

## Introduction and background

Sleep hygiene has been a topic of interest for researchers who are studying short- and long-term effects of sleep disorders and sleep deprivation in children. Children who regularly receive an adequate amount of sleep, both in quantity and quality, overall display improved attention, memory, behavior, mental and physical health. Over a period of time, these same children can present with hypertensive issues, risk of obesity, and chronic depression [1]. 

In this review article, we will pay particular attention to environmental factors that can negatively impact a child’s sleep cycle and ultimately health-specifically, electronic devices such as mobile devices, portable tablets, and television. As the years have progressed, technology has advanced. More electronic devices are now found in the average household with a third of Americans living in households with at least three smartphones. In 2016, approximately 70% of Americans owned a tablet, 80% possessed at least one computer, nearly 90% owned at least two smartphones, and 90% of US adults owned approximately five electronic devices per household [2]. Throughout the years, schools have been integrating this technology into their daily lectures. Research projects and homework often rely solely on internet access and possession of an electronic device. In most recent times, due to the 2020 coronavirus pandemic, while many education centers were physically temporarily suspended, a transition to virtual learning commenced. Along with restrictions in many parts of the world preventing citizens from spending much time outdoors, the increased use of electronic devices in children has led to poor sleep habits as well as other behavioral and lifestyle complications, particularly when used during late hours of the evening when physiologically, the body is preparing itself for nocturnal rest and recovery.

Our bodies are structured around a 24-hour “body clock” referred to as a circadian rhythm. For each hour we are awake throughout the day, our bodies naturally develop a drive for sleep which is regulated through a naturally secreted hormone, adenosine. This adenosine continues to build in our brain until our body naturally shifts to desired sleep. While we are asleep, our body works to decrease the amount of adenosine and properly trigger an awakening in the morning hours with the increase in another hormone known as cortisol. There is also a peak of yet another hormone, melatonin, which increases in our body in response to darkness. As humans age, their need for sleep decreases. Infants can sleep anywhere from 12-16 hours daily (including naps), while adults over the age of 18 can function efficiently with seven to eight hours of sleep daily [3]. In Table 1 below, the recommended hours of sleep based on specific age in children is a recommended guideline to keep in mind as we discuss sleep deficiency and how sleep deprivation can impact the normal number of hours recommended for body recovery and function.

**Table 1:** Recommended duration of sleep (in hours) based on age, as per Johns Hopkins All Children’s Hospital.

Age	Recommended Duration of Sleep (hours)
Infants (<1 year of age)	12-16 hours
1-2 years of age	11-14 hours
3-5 years of age	10-13 hours
6-12 years of age	9-12 hours
13-18 years of age	8-10 hours

As mentioned earlier, our melatonin levels are dependent upon the environmental darkening of our environment during the night time hours. By introducing electronic devices such as laptops, tablets, smartphones, and television screens just prior to sleep, many children and adults are interfering with the natural hormone production and regulation. Melatonin levels fail to increase, and so, leads to a false “awakened” state which resembles how the brain normally functions during the daytime hours.

This article aims to explore the detrimental short-term and long-term negative effects of sleep deficiency in children, specifically induced by environmental triggers such as light-emitting electronic devices. This article will evaluate the quality and quantity of sleep, as well as other sleep deprivation-induced associated chronic conditions which may ultimately alter the course of a child’s life.

## Review

Methods

PubMed, Google Scholar, PewResearch, Johns Hopkins School of Medicine, and ResearchGate online databases were exclusively used for purposes of collecting corresponding data. Of the 20 scientific papers yielded, specific inclusion and exclusion criteria were applied. After inclusion and exclusion criteria were applied, 11 scientific papers were included in our final review. All 11 of those papers met the specifications and were peer-reviewed.

Inclusion and exclusion criteria

Each of the scientific papers included in our final review was composed in English and included data collected and reviewed from 2005-2019. All cases discussing the effects of electronic use and affected sleep in children were conducted through surveys. The included scientific papers contain data collected from large sample sizes in a particular geographic area. All test subjects included in the various surveys were of approximately similar age. Several of the scientific reviews which met inclusion criteria used the Pittsburgh Sleep Quality Index (PSQI), the Fatigue Assessment Scale (FAS), and the Bergen Insomnia Scale (BIS) in order to assess daytime sleepiness and insomnia. Articles that were excluded from this review lacked sufficient sample sizes and did not fall into the category of environment-induced sleep disorders.

Results

Of the six scientific papers, all demonstrated a correlation between late-night electronic use and a decrease in rapid eye movement (REM) sleep and latency. A study conducted in two clinics, the Penn State General Pediatrics Clinic and the Penn State Specialty Clinic for Childhood Obesity, conducted on patients between the ages of 8 and 17, with parental permission, assessed six domains-electronic device use patterns, child sleep, attention/focus, nutrition habits, activity levels, and the electronic device used amongst their parents. The child’s body mass index (BMI) was also evaluated during the span of the study. Two-hundred and seven surveys were completed and evaluated for this study. Results showed children who watched television before bedtime and those who used their smartphones before bedtime were more likely to be overweight/obese. There was no association between BMI and the use of the computer. On average, children who watched television slept 30 minutes less on average, and those who used their smartphones slept an hour less than average. Children who used computers at bedtime also slept an average of one hour less and also had trouble falling asleep. Children who used a computer and their smartphones before bed woke up fatigued and denied breakfast. Television use for some reason did not result in morning fatigue, however, did result in denial of breakfast. Playing sports outdoor for at least four hours daily significantly decreased the risk of obesity in all age groups. Those children with a higher BMI were also at higher risk of being diagnosed with depression, attention deficit hyperactive disorder (ADHD), and asthma. There was no correlation between parental electronic device use amounts and child electronic device use amounts [4].

In a study conducted in Alberta, Canada, a sample of 3,398 grade 5 students were administered the Harvard Youth/Adolescent Food Frequency questionnaire aimed at assessing physical activity, height/weight and the impact of night-time access to the use of electronic devices and sleep, diet quality, physical activity, and overall BMI. Approximately 64% of those fifth-grade children had access to one or more electronic device in their bedroom. Those with nighttime access all shared similar characteristics of poor sleep quality in terms of duration, poor dietary habits, obesity, and overall lower physical activity levels--all within statistically significant values [5].

In a 2007 study published in the American Academy of Pediatrics, eleven school-aged children in Germany were recruited for a polysomnographic study that exposed these children to voluntary excessive television and computer gaming. In addition to this sleep study, a visual and verbal memory test was administered before and after brain stimulation. While there were no effects on REM sleep, television, and video games prior to sleep reduced sleep efficiency and prolonged sleep onset. Verbal cognitive performance and memory deteriorated [6].

In Norway, a cross-sectional population-based survey of 9,846 adolescents from 2012 compared the effects of daytime electronic device use and night-time electronic device use to sleeping patterns. All candidates in this trial were between the ages of 16 and 19. Results showed in both instances of electronic device use that there was a shortened period of sleep duration, long sleep onset, and sleep deficiency. The average acceptable amount of sleep, seven to eight hours nightly, was seen to be much lower in computer users just prior to bedtime, five hours [7].

A sample of 844 teenagers and adults ranging from 18-94 years old in Belgium were studied using the Pittsburgh Sleep Quality Index (PSQI), forward associative strength (FAS), and backward associative strength (BAS) indexes to determine how bedtime electronic device use affects various qualities of sleep. Half of the individuals owned a smartphone device, and 60% of those individuals used their mobile devices in their bedrooms. All individuals experienced longer sleep latency, poor sleep efficiency, increased daytime dysfunction, and insomnia. The majority of the symptoms were expressed in individuals less than 42 years old, while in those above the age of 60, shorter sleep duration and earlier rise time was experienced [8].

In a 2014-2015 study conducted in Geneva, Switzerland, five-hundred sixty-nine participants ranging from 12 to 19 years old were surveyed about their night-time electronic device use and the impact it has on their sleep. These participants also wore actimeters in order to statistically graph data during their sleep periods. Participants were also advised to avoid caffeine-containing products and sour foods after 3 pm. Results showed that screen use during evening hours not only delayed sleep onset, however, it also shortened sleep duration, particularly in those participants in high school. It was noted that high school students slept longer hours during the weekend in order to compensate for the lack of sleep they experienced throughout the week due to the average nightly 79 (+/- 3) minute average electronic device use prior to falling asleep. Melatonin samples were taken from 13 participants in the form of saliva testing. All 13 cases showed delayed onset in rising of melatonin levels in those participants with night time electronic device use [9].

The six scientific studies researched in particular for this scientific review article are organized in Table 2, below. Each study is categorized by the location in which they were conducted, along with the sample size and the conclusions drawn within each study. 

**Table 2:** Description of selected studies that met inclusion criteria for this review.

Study	Location	Study Period	Sample Size	Conclusion
Fuller, et al. [4]	United States	March 2016-September 2016	207 (8-17 years old)	Electronic device use before bed time increased inattentiveness, risk for obesity, and decreased sleep quality and duration. Parental use of electronic devices did not impact amount of child use of electronic devices.
Chahal, et al. [5]	Canada	2012-2013	3,398 (5^th^Grade Students)	Over 60% of children had access to at least one or more electronic device in their bedroom. Access to night time use of these electronic devices resulted in shortened sleep duration, excess body weight, poor diet, and physical inactivity.
Dworak, et al. [6]	Germany	2007	11 (school-aged children)	Television viewing reduced sleep efficiency, but did not alter sleeping patterns. Computer gaming prolonged sleep onset and reduced slow-wave sleep as well as verbal memory performance.
Hysing, et al. [7]	Norway	2012	9,846 (16-19 years old)	Daytime and bedtime use of electronic devices and increased screen time decreased sleep duration, increased sleep latency onset, and increased the risk of sleep deficiency. Computer users often risked less than 5 hours of sleep compared to the average 7-8 hours.
Exelmans, et al. [8]	Belgium	2015	844 (18-94 years old)	Bedtime mobile device use not only negatively affects the youth, but also adult sleeping patterns. The younger population spent more time in comparison to adults on mobile smartphones prior to sleep, thus directly impacting the youth more.
Perrault, et al. [9]	Switzerland	November 2014-May 2015	569 (12-19 years old)	Actigraphy & Salivary Melatonin Sampling resulted in decreased sleep duration and quality of sleep throughout the weekdays and compensatory oversleep on weekends to recover. Slower than average rising of melatonin levels in those using nighttime electronic devices emitting light.

Limitations

Several scientific papers failed to depict an environmental contributor to poor sleep quality expressed in children. Many of the children were not evaluated for any chronic or underlying genetic predispositions to sleep disorders. Other studies were conducted over two decades ago when such technology described in our scientific review may not have been readily available to the majority of children and/or households. 

Discussion

As discussed previously, with each year that passes, households are acquiring newer more advanced technological devices, which more often than not, are portable and easily accessible. The majority of households across the globe have more than a single smartphone, laptop/computer, tablet, television screen. Just as everything else in life, owning and operating an electronic device comes with responsibilities. Children and adults in the studies researched had access to daytime and nighttime electronic devices prior to bedtime. This decision directly impacts their sleep cycles and patterns, sleep latency, sleep duration, and behavior. When discussing long-term effects, chronic and constant technological abuse during late evening hours has been linked to obesity, hypertension, psychologic and neurocognitive delays-such as social anxiety disorders, depression, and ADHD. 

Social Anxiety and Depression

In the recent studies visited, regardless of the type of electronic device in question and age, the majority of participants in the various studies suffered from an increased sleep onset and a decreased duration of sleep. A study conducted in 2018 determined that excessive night time internet use, including but not limited to social messaging, television, and gaming contributed to emotional vulnerability and dysregulation due to the interruption of physiologic sleeping patterns, ultimately resulting in a high risk of depression. 

Having technology at arm’s length throughout the night creates a nonverbal interaction between multiple individuals at a click on a button. This use of technology may stun a child’s ability to socially and verbally interact with their peers on a personal level, as they enter a virtual comfort zone. Due to this instant response rate seen with messaging through electronic devices, many children lose quality, duration, and efficiency of sleep as they persist on sleeping next to devices that emit light and sound. Social distancing and overuse of technology particularly at night may lead to difficulties with constructing and maintaining relationships and friendships, as well as maintaining high academic performance. 

ADHD: Hyperactivity and Impulsivity

Up to 10% of the world’s population suffers from ADHD. This is a problematic condition linked to impulsivity, hyperactivity, and a lack of an attention span. This condition in severe cases may involve children seeking relief and a decrease in anxiety through the use of technology and online interactions. This stimulation they receive through electronic devices, along with the decreased quality of sleep due to hyperactivity can pose a threat to long term mental health in these children [10,11].

## Conclusions

While there are many factors contributing to unhealthy habits and lifestyles in children and young adults, night time electronic device use and any other light-emitting sources may create a harmful environment and a tumbling vicious cycle of poor habits leading to an unhealthy lifestyle to develop. In summary, in >90% of studies conducted worldwide, including those which were excluded from this scientific paper, an increased amount of screen time directly correlated with delayed onset of sleep and decreased overall amount of necessary sleep for body and brain recovery and rejuvenation. When comparing computer use to television use, poorer sleep outcomes resulted from computer use, most likely due to the ability to interact with computers. Parents cannot consistently control their child’s nighttime screen time; however, measures are strongly urged for parents to start their children on a disciplined night time routine which involves an electronic device curfew at a young age. This can aid in establishing a healthy foundation for years to come. This curfew may also extend in a “lights out” approach to ensure their child is tucked away in bed at a reasonable hour with the lights shut off in order to ensure the rise of melatonin levels and the decline of adenosine levels. 
